# Decision Makers Use Norms, Not Cost-Benefit Analysis, When Choosing to Conceal or Reveal Unfair Rewards

**DOI:** 10.1371/journal.pone.0073223

**Published:** 2013-09-16

**Authors:** Marco Heimann, Vittorio Girotto, Paolo Legrenzi, Jean-François Bonnefon

**Affiliations:** 1 Institut d'Administration des Entreprises and Laboratoire Cognition Langues Langage Ergonomie, University of Toulouse, Toulouse, France; 2 Dipartimento di Culture del progetto, University IUAV of Venice, Venice, Italy; 3 Dipartimento di Filosofia e Beni Culturali, University Ca' Foscari Venice, Venice, Italy; 4 Laboratoire Cognition Langues Langage Ergonomie, University of Toulouse and Centre National de Recherche Scientifique, Toulouse, France; Brain and Spine Institute (ICM), France

## Abstract

We introduce the Conceal or Reveal Dilemma, in which individuals receive unfair benefits, and must decide whether to conceal or to reveal this unfair advantage. This dilemma has two important characteristics: it does not lend itself easily to cost-benefit analysis, neither to the application of any strong universal norm. As a consequence, it is ideally suited to the study of interindividual and intercultural variations in moral-economic norms. In this paper we focus on interindividual variations, and we report four studies showing that individuals cannot be swayed by financial incentives to conceal or to reveal, and follow instead fixed, idiosyncratic strategies. We discuss how this result can be extended to individual and cultural variations in the tendency to display or to hide unfair rewards.

## Introduction

Imagine that you had a fancy dinner with friends, splitting the bill among you all. Later on, you discover that your own check was never cashed, meaning that you (and you only) ate for free that night. Would you tell the story to your friends, or would you keep quiet about it? Or imagine that, just as former German President Christian Wulff, you benefited from a legal but unusually low-interest loan. Would you let the voters know, or would you try to stop the story from being published? Christian Wullf allegedly did the latter, and the subsequent public outcry led to his resignation in February 2012.

These two examples illustrate what we call the Conceal or Reveal Dilemma: the decision to hide or to disclose that one got something which others would want for themselves, and that it happened for no good reason. Our contention in this article is that individuals make this decision following idiosyncratic norms, rather than by rational cost-benefit calculations, or by the application of a universal norm. Critically, we report four experiments showing that individuals do not respond to financial incentives in a paradigm that we call the Conceal or Reveal Game, and that they are split in terms of the norm they apply to the situation.

### 0.1 The Conceal or Reveal Dilemma

We consider that an agent 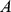 faces the Conceal or Reveal Dilemma when she receives a benefit with two characteristic features: secrecy and unfairness. *Secrecy* means that no other agent knows about the benefit unless they are the ones who intentionally provided it. *Unfairness* means that whatever agent 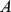 thinks of the deserved or undeserved nature of the benefit, she knows that others are likely to see the benefit as unfair if they learn about it. Agent 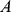 must choose between two options: keeping the benefit a secret (the Conceal option), or letting other agents know about it (the Reveal option).

#### 0.1.1 Cost and benefit resolution

The standard, decision-theoretical approach to the Conceal or Reveal Dilemma would be for a rational decision maker to weight the expected benefits and costs of the two options.

On the one hand, revealing an unfair benefit is likely to trigger negative reactions from other agents, such as malicious envy and retorsion measures. People (but also dogs and monkeys [1], [2]) react negatively to unfairness in reward distributions, and they might impose all sorts of penalties to agents who enjoy undeserved benefits [3]–[5].

On the other hand, a decision to conceal an unfair benefit comes with the risk of being discovered and perceived as a liar. A reputation as a deceiver can result in a broad range of specific costs, such as intensely negative reactions from others [6], aggravated third-party punishment [7], and fewer opportunities to join partners coalitions [8]. Another risk, rare but real, is to be targeted for blackmail by unscrupulous agents [9].

Even from this cursory analysis, it is immediately apparent how difficult it is to optimize in the Conceal or Reveal Dilemma. One will find it difficult to think of all possible outcomes (e.g., blackmail), to translate outcomes in utility points (e.g., missed opportunities to join coalitions), and to assess the probabilities of the various outcomes (e.g., third-party punishment conditional on discovery). These three features are precisely that identified [10] as conducive to another form of decision-making, that is, the use of deontic norms. We now turn to this alternative resolution of the dilemma.

#### 0.1.2 Deontic norm resolution

As an alternative to the use of cost-benefit analysis, the Conceal or Reveal Dilemma can be resolved by relying on deontic norms, which only consider the intrinsic acceptability of the two possible actions (conceal, reveal), and do not factor in their consequences. Recent research emphasized the importance of these deontic norms for moral decision making [11], and especially their use as substitutes to cost-benefit analysis [8], [10], [12], [13].

Deontic norms can provide a workable alternative to cost-benefit analysis, when it is hard (or plain impossible) to assess the range of potential outcomes, to translate them into utility points, and to assign them each a probability [10]. With respect to the Conceal or Reveal Dilemma, a deontic resolution consists of applying a general norm supporting one option or the other, regardless of the consequences of this option.

An important aspect of the Conceal or Reveal Dilemma is that it does not lend itself to the application of any universal (or quasi-universal) deontic norm such as *do not inflict harm*. Indeed, no harm is done either way, financial or otherwise. None is made poorer or richer as a consequence of the decision to conceal or to reveal. Different norms can be considered instead, that would either support concealing or support revealing. For example, one deontic reformulation of the Conceal or Reveal Dilemma is *do not boast* vs. *do not lie*. Boasting about one's undeserved rewards would amount to advertising a violation of one of the fundamental moral motives, that of proportionality of merit and reward [14]. On the other hand, concealing the reward would amount to lying, and there is evidence that a substantial proportion of people prefer not to lie, even if it means they will incur a cost [15]–[19].

The fact that concealing and revealing can both be supported by (different) deontic norms is important because it makes it impossible to predict, ex ante, what people are going to do. The deontic norm model makes a critical prediction, though, that distinguishes it from the cost-benefit model: It predicts that whatever people decide to do, they will not change in response to financial incentives. In contrast, if people solve the dilemma by cost-benefit analysis, they should be swayed by experimental manipulations that make it costly or beneficial to conceal or to reveal. In this article, we provide a detailed test of these critical predictions.

### 0.2 The Conceal or Reveal Game

We used the Conceal or Reveal Game in four experiments. Experiment 3 was run on campus for real incentives, and with a real opponent in the game. The other experiments were run online using Mechanical Turk. The game proceeds as follows:

Two players compete in a quiz game for a prize (1 or 2 euros, depending on the experiment). The winner (the player with the greater number of points in the quiz) gets the prize as promised. However, the loser is secretly offered a choice. She is to get a special bonus, whose value depends on whether she decides to conceal or to reveal the bonus. The two options read as follows (minus the Conceal and Reveal headers):

**Conceal** You get 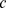 euros and we do not tell it to the other player, so the other player will not know that you earned money while you lost the game. That is, you get 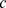 euros and the final result we will show to the other player will be: 'You scored more points than your opponent. Your reward is [amount of the prize], and the reward of your opponent is 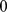 euros.”

**Reveal** You get 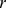 euros and we tell it to the other player, so the other player will know that you earned money while you lost the game. That is, you get 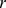 euros and the final result we will show to the other player will be: 'You scored more points than your opponent. Your reward is [amount of the prize], and the reward of your opponent is 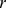 euros.”

In all the experiments we report, 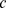 and 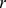 were greater than the amount of the prize going to the winner in the quiz game. Our main variable in all experiments was the difference between 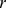 and 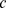, that is, the financial incentive to reveal the bonus. The greater 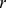 compared to 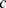, the higher the incentive to reveal; and the smaller 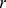 compared to 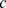, the greater the incentive to conceal.

## Methods

### 0.3 Ethics statement

Apart from the online data collection on MTurk, data were exclusively collected at the University of Toulouse-2, France. As of the date of data collection and manuscript submission, written consent was not required by the University of Toulouse-2 where the research was conducted. Accordingly, participants verbally provided informed consent to participate in the study, a process which was monitored by the first author. Ethics approval by the “comit de protection des personnes” (under Loi Huriet-Srusclat 881138) being a legal requirement for biomedical research only, it was not required for a basic behavioral study such as ours. Accordingly, current regulations dispensed us from seeking approval from the “comit de protection des personnes” when we collected these data. All participant data were anonymised.

### 0.4 Experiments 1–2

The experiments were conducted on the Mechanical Turk platform. Subjects read a narrative describing their hypothetical progression in the Conceal or Reveal Game (subjects got to answer two trivia questions to illustrate the quiz part of the game). They indicated whether they would choose the Conceal option or the Reveal option, if the game was performed for real. Note that since the game was hypothetical, we do not know whether subjects would have won or lost in a real quiz – which rules out the possibility of having selected people with poor general knowledge, or other individual traits associated with a poor performance at a trivia quiz. There were 120 participants in Experiment 1 (46 women, mean age 30), and 219 participants in Experiment 2 (96 women, mean age 32). The incentive to reveal (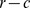) was manipulated between-subject. It was 1, 2, or 3 euros in Experiment 1 (in which 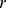 was kept constant at 5 euros), and 1 or 5 euros in Experiment 2 (in which 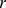 was kept constant at 6 euros).

After they made a choice, participants indicated the extent to which they thought the other player would envy their purported payoff, using a 4-item scale [20]. This manipulation check allowed us to test whether our manipulation of incentive made a subjective difference for the participants; that is, whether participants themselves made a difference in the utility of winning 1, 2, 3, or 5 euros, by attributing more envy to their counterpart as a function of the earned sum.

### 0.5 Experiment 3

The experiment was conducted on the campus of the University of Toulouse (France), with 240 participants (94 women, mean age 23). Participants were explicitly told that all financial gains were for real, and that the experimenters might decide to award discretionary bonuses during the game. Participants were paired up to compete in the 8-question trivia quiz. Within each pair, the participant with the lower score (the quiz loser – ties were resolved through a supplementary list of questions) secretly received the Conceal or Reveal offer. The 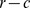 incentive to reveal was manipulated between-subjects, and could be −1, 0, +1, or +8 (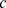 was kept constant at 2 euros). While the quiz loser was considering the Conceal or Reveal offer, the quiz winner was given a personality questionnaire to fill. This was done in order to keep both players busy, and to avoid a situation where the quiz loser would be under scrutiny from the quiz winner while making a decision.

Once payments were given, quiz losers indicated the extent to which they thought the quiz winner envied them (same 4-item scale as in Experiments 1 and 2), and quiz winners indicated how much they actually envied the quiz loser (same 4-item scale). This procedure provided further opportunity to check whether our manipulation of incentives made a subjective difference for the participants, both winners and losers.

### 0.6 Experiment 4

The experiment was conducted on the Mechanical Turk platform, exactly as in Experiments 1 and 2, except that the 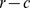 incentive was manipulated within-subject. The 330 participants (143 women, mean age 29) made five Conceal or Reveal decisions (in randomized order), with 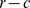 incentives of −8, −1, 0, +1, and +8 (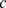 was kept constant at 10 euros). To keep the experiment reasonably short, the 4-item envy scale was omitted.

## Results

**Figure 1:**
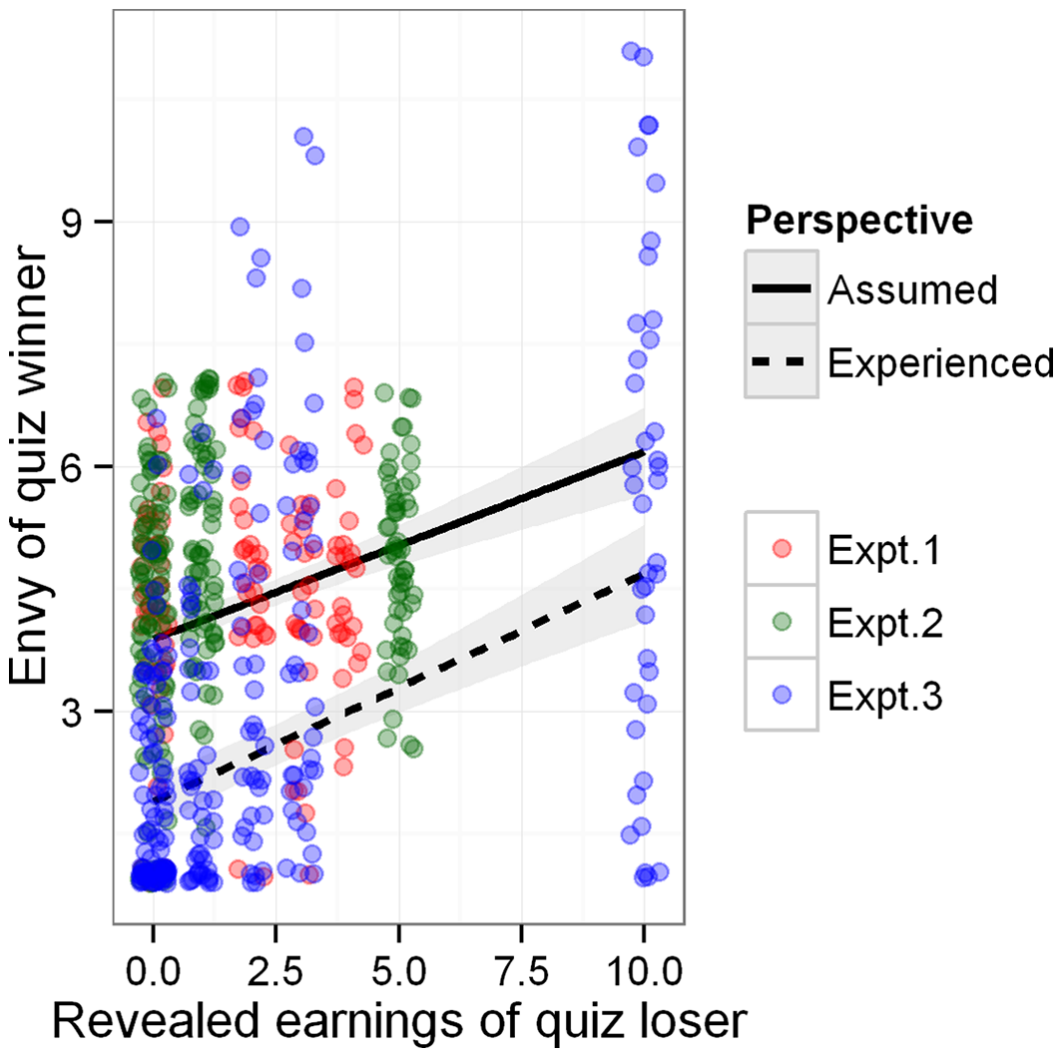
Participants (quiz losers) correctly assume others (quiz winners) to envy them all the more than their revealed earnings are higher. They slightly overestimate this experienced envy.

Our manipulation checks showed that the incentive manipulation itself was successful. Figure 1 shows that the participants expected others to envy them the more the higher their revealed earnings were. They were correct indeed, as shown by the data of Experiment 3, in which we had an opportunity to measure the actual envy that other players experienced. If anything, participants slightly overestimated the envy they were occasioning to others.

Statistics confirm the visual impression produced by Figure 1. Predicted envy was correlated with the earnings of the quiz loser in Study 2 and 3, respectively 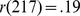, and 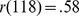 (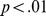, 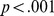); albeit not in Study 1: 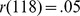 (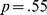). Finally, as measured in Study 3, experienced envy was correlated with the earnings of the quiz loser, 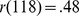, 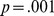, and predicted envy was significantly higher than experienced envy, 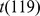, 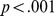, 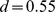.

Having shown that we successfully manipulated incentives, we turn to the effect of these incentives to the Conceal or Reveal Decision. Table 1 displays the proportion of participants who chose to Reveal, for all levels of incentives across our five experiments. It is immediately apparent that in all but one of our 14 experimental conditions, a majority of participants chose to Reveal (even in the three conditions in which it was costly to do so).

**Table 1:** Percentage of participants choosing to reveal, in all experiments, as a function of the incentive to reveal (in euros).

Incentive	−8	−1	0	+1	+2	+3	+5	+8
Expt. 1				72	57	62		
Expt. 2				59			51	
Expt. 3		70	60	60				66
Expt. 4	54	58	60	48				53

Increased incentives to reveal did not increase the proportion of participants choosing to reveal, in any experiment, and even when the incentives were real. Since outcome was dichotomous, logistic regression models were used for analysis (cf. Table 2). Generalized linear models did not detect any effect of incentives in Study 1, Study 2, nor Study 3. Neither did a mixed effects model used to account for fixed effects in Study 4.

**Table 2:** Parameter estimates of logistic regression models for Experiment 1, 2, 3, and a linear mixed model for Experiment 4.

	*Experiment* 1	*Experiment* 2	*Experiment* 3	*Experiment* 4
(Intercept)	1.53 (0.77)*	−0.12 (0.68)	0.15 (4.14)	0.53 (0.16)
−8				−0.27 (0.18)
0			−0.47 (0.55)	0.10 (0.19)
+1			−0.46 (0.55)	−0.60 (0.18)
+2	−0.66 (0.48)			
+3	−0.47 (0.50)			
+5		−0.32 (0.41)		
+8			−0.21 (0.57)	−0.30 (0.18)
Quiz score			0.06 (0.13)	
Age	−0.01 (0.02)	0.02 (0.02)	0.00 (0.05)	−0.00 (0.02)
Male	−0.49 (0.40)	−0.52 (0.44)	0.17 (0.41)	−0.32 (0.39)
AIC	162.49	140.62	169.09	2024.13
BIC	176.43	150.96	188.60	2056.58
Log Likelihood	−76.25	−66.31	−77.54	−1006.07
Deviance	152.49	132.62	155.09	2012.13
Num. obs.	120	98	120	1650
Num. groups: ID				330
Variance: ID.(Intercept)				2.91

***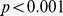, **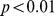, *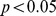.

In four studies we did not detect a significant effect of incentives on the proportion of participants choosing to reveal. Therefore, it is appropriate to compute the meta-proportion of reveal decisions, across our four studies.

**Figure 2:**
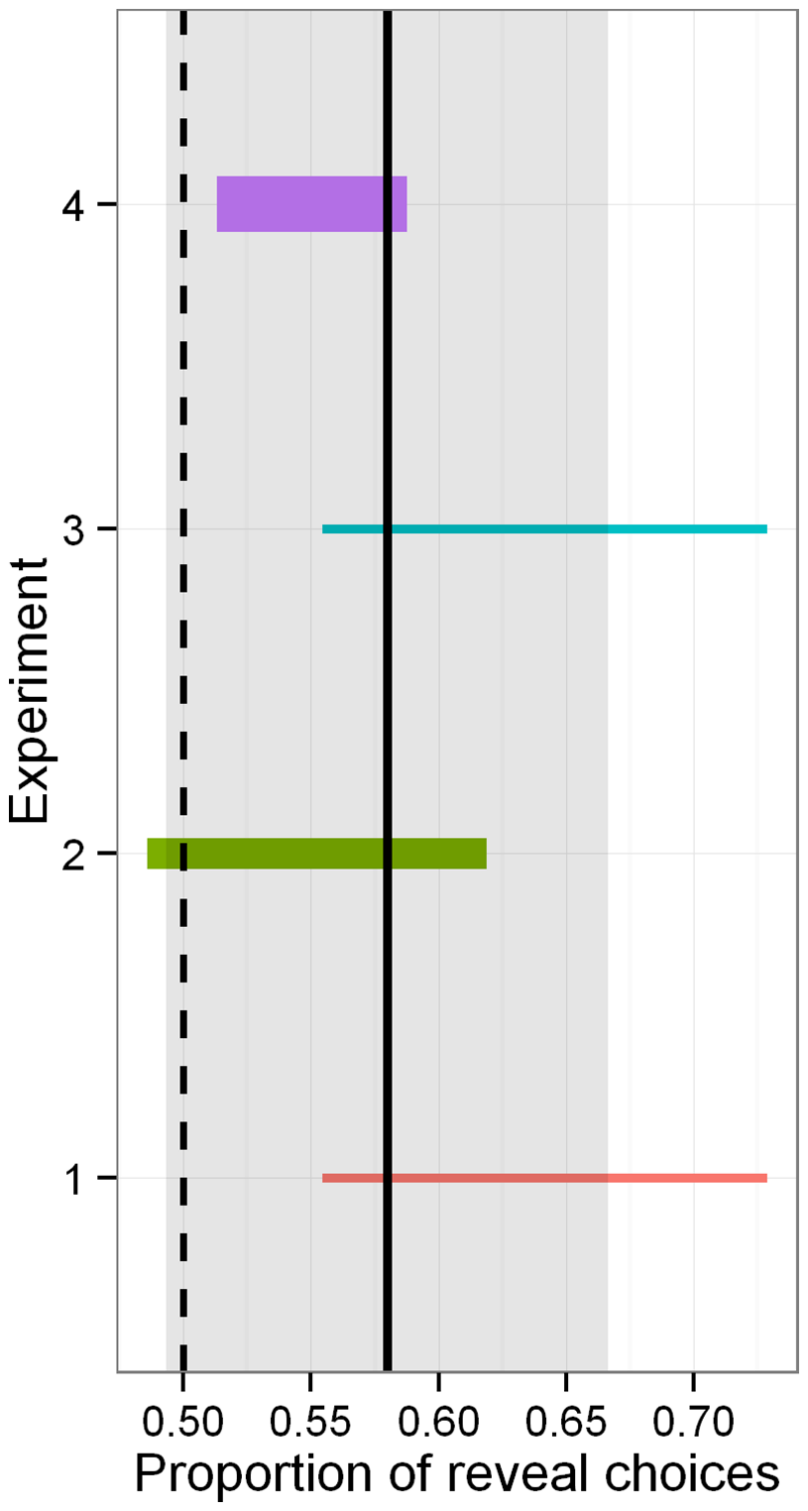
Meta-proportion analysis of Studies 1–4. Line width is proportional to Study 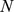, line length shows confidence interval of the proportion of participants deciding to reveal. The vertical dark line displays the meta-proportion across studies, surrounded by its confidence interval in gray.

Figure 2 offers a visual display of this analysis. Across our four studies (totalling over 800 participants), the proportion of reveal decision is estimated at. 58, with a 95%- confidence interval of .54–.62, p ¡ 0.001. Further, even a permissive test does not detect any difference between the eight incentive conditions, 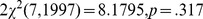.

## Discussion

Our experiments put people in an uncomfortable situation, that of making public (or not) that they received unfair benefits. It turned out that about 60% of subjects preferred to advertise their unfair benefits, whatever the personal incentives or counterincentives to do so. The incentives we offered were modest (about 10 euros at most), but in the same range as most studies of dishonesty [18], [21], as well as a vast number of behavioral economics experiments [22], [23].

This insensitivity to incentives is a characteristic sign that a decision is the product of a norm, rather than a cost-benefit analysis. In that respect, a critical feature of the Conceal or Reveal Dilemma is that it does not simply oppose a morally commendable decision to a less commendable one. In particular, subjects cannot change the fact that their reward is unfair, and have no way to behave altruistically in favour of the other player. That is, if they stick to the implicit rule of non-transferable utility. In one (and only one) of the 120 pairs of Study 3, did a subject thought of publicly accepting 10 euros, then sharing them with the other player. The absence of a strong, straightforwardly applicable norm, paves the way for cultural and individual variations in response to the Conceal or Reveal Dilemma.

### 0.7 Cultural variations

The norms that individuals can apply in the Conceal or Reveal Dilemma do not appear to be strong and universal (and different individuals might even apply different norms for the same final decision). The cultural prevalence of different norms could accordingly lead to cultural differences in response to the Conceal or Reveal Dilemma.

For example, it seems possible that a norm of modesty might encourage the decision to conceal, given that modesty personality scales often include items related to the avoidance of attention-seeking [24], phrased for example as *I don't call attention to myself* or *I dislike being the center of attention* (see the Modesty/Humility scales of the Values in Action, NEO Personality Inventory, and HEXACO Personality Inventory, all available from ipip.ori.org). Interestingly for our current purpose, there are known cultural variations in the importance of the modesty norm. For example, the modesty norm is substantially stronger in some collectivist cultures [25], [26], and the effects of this cultural stricture can be detected early on. For example, Chinese children judged modest lies more positively and boastful truths less positively than Euro-Canadian children, a cultural difference which was shown to increase with age [27].

It is thus quite possible that cultural differences in the strength of the modesty norm might translate in differences in the frequency of concealing decisions in the Conceal or Reveal dilemma. Other cultural differences, for example in the likelihood of self-disclosure, might have an impact as well on Conceal or Reveal decisions: it will be an important task in the future to map cultural differences in relevant norms onto behavioural differences in the Conceal or Reveal Dilemma.

### 0.8 Individual variations

Whatever the (counter) incentives, about 60% of subjects in our experiments decided to reveal their benefits. There are two possible interpretations of this finding, that speak directly to current debates in moral-economic decision making research. In a nutshell, either individuals follow a strict norm when they face the Dilemma (and for 60% of them, the rule is to Reveal), or individuals randomly make a decision every time they face the dilemma, with 60–40 odds in favor of Revealing. In other words, either the Conceal and Reveal Game elicits a mixed population, or it elicits a mixed strategy.

Recent research on cheating would speak for the mixed strategy hypothesis [19], [21], [28]. The frequency of cheating seems to be stable whatever the incentives to cheat, but this stability is not due to some individuals being systematic cheaters and others being systematically honest. Rather, it reflects the fact that everybody cheats a little. The stable frequency of cheating seems to reflect a mixed strategy, rather than a mixed population. The bulk of the literature on moral-economic decision making would nevertheless favor the mixed populations hypothesis. The first and foremost framework for explaining insensitivity to incentives in moral-economic decision making is that of *sacred* or *protected* values [29]–[32]. These values correspond to core elements of one' identity (be them religious, ethnic or otherwise), and their characteristic feature is to resist tradeoffs. Typically, one will refuse to transgress a sacred value for money, and will even get upset if asked for one's price. One could tentatively interpret incentive-insensitivity in the Conceal or Reveal Game as the sign that sacred values are at work, and thus that the 60–40 split reflects a mixed population. It is slightly odd, though, to think that concealing one's unfair benefits could be a sacred value for 40% of the population.

There is another framework that would speak for the mixed population hypothesis, without appealing to sacred values. In the mutualistic model of morality [8], decisions are made to optimize one's future participation in profitable coalitions, by means of establishing a reputation as a decent partner. Baumard and colleagues argue that this optimization is more likely to be reached by agents who evolved genuine moral preferences, than by agents who evolved to compute the expected costs and benefits of each moral decision. This evolutionary model would predict again that the 60–40 split reflects the evolution of a mixed-population equilibrium in the Conceal or Reveal Game. A natural direction for future research, in order to arbitrate between the mixed-population and mixed-strategy accounts, would be to develop evolutionary game-theoretic models of the Conceal and Reveal Game (that would include, e.g., third-party punishment and meta-punishment [33]), and to check the conditions under which the equilibrium supports mixed populations or mixed strategies.

From an experimental perspective, though, our data already point in one direction. Experiment 4 followed a within-subject design, in which participants made a series of five decisions, under various levels of incentives. Our data suggest that individuals adopted one strategy and stuck to it for all levels of incentives. About 38% of subjects made the same choice in all five situations, and 65% made the same choice in four situations out of five. While these data are only suggestive, they should orient future research towards the possibility that individuals have evolved *genuine* but *different* moral preferences about what to do in the uncomfortable situation of having been granted unfair benefits.
